# Thyroid dysfunction in children and adolescents affected by undernourished and overnourished eating disorders

**DOI:** 10.3389/fnut.2023.1205331

**Published:** 2023-09-29

**Authors:** Valeria Calcaterra, Vittoria Carlotta Magenes, Francesca Siccardo, Chiara Hruby, Martina Basso, Veronica Conte, Giulia Maggioni, Valentina Fabiano, Susanna Russo, Pierangelo Veggiotti, Gianvincenzo Zuccotti

**Affiliations:** ^1^Department of Internal Medicine and Therapeutics, University of Pavia, Pavia, Italy; ^2^Department of Pediatric, Buzzi Children's Hospital, Milan, Italy; ^3^Child and Adolescent Neuropsychiatry Unit (UONPIA), ASST-Fatebenefratelli-Sacco, Milan, Italy; ^4^Department of Biomedical and Clinical Science, University of Milano, Milan, Italy; ^5^Pediatric Neurology Unit, Buzzi Children's Hospital, Milan, Italy

**Keywords:** eating disorders, children, adolescents, anorexia nervosa, binge eating disorder, bulimia nervosa

## Abstract

Eating disorders (ED) are one of the most prevalent chronic disorders in adolescents and young adults, with a significantly increasing prevalence in younger children, particularly in girls. Even if obesity in essence is not framed as an eating disorder and has always been considered a separate pathology, ED and obesity could be considered part of a continuum. It has become evident that one condition can lead to another, such as binge eating disorder (BED) and bulimia nervosa, and that they share the same repercussions in terms of psychosocial, metabolic, and nutritional health. This narrative review aims to investigate the hypothalamic-pituitary-thyroid axis in undernourished and overnourished patients with ED, including obesity, in order to highlight the relationship between weight control and thyroid function and its effects and to consider therapeutic and preventive strategies in children and adolescents. Literature data report that thyroid alterations occur in patients with ED, both underweight and overweight, and represent a continuum of changes depending on the severity and time course of the disease involving the endocrine system. Considering the relevant role thyroid hormones (TH) play not only in energy expenditure (EE) but also in metabolic control and cardiovascular risks related to dysmetabolism and mood regulation, continuous monitoring of thyroid homeostasis in patients with ED is mandatory to prevent severe complications and to start early treatment when necessary.

## 1. Introduction

Eating disorders (ED) are one of the most prevalent chronic disorders in adolescents and young adults, with a significant growing prevalence in younger children (1–3). These disorders are more prevalent in women, but, their prevalence has also increased in men and minority groups in recent years. Abnormal eating or weight-control behaviors are the core symptoms of ED (4–6). Despite their high prevalence, these disorders often remain underdiagnosed, leading to a chronic and severe course (6–8).

In parallel, being overweight and obese during childhood represents serious health issues (9). Indeed, the prevalence of obesity has increased worryingly over the last three decades, reaching epidemic proportions worldwide, most notably in Mediterranean countries. In Italy, ~20 and 9% of the children presented as overweight or obese in 2019, respectively (10–12). The pathogenesis of obesity is multifactorial, including genetic, epigenetic, environmental, sociocultural, physiological, and various other factors that contribute to the origin and persistence of this condition (13). Moreover, obesity remarkably affects the quality of life of the affected patients and is associated with the risk of premature death and significant comorbidities, including adverse effects on physical and psychosocial health (14–16).

**Figure 1:**
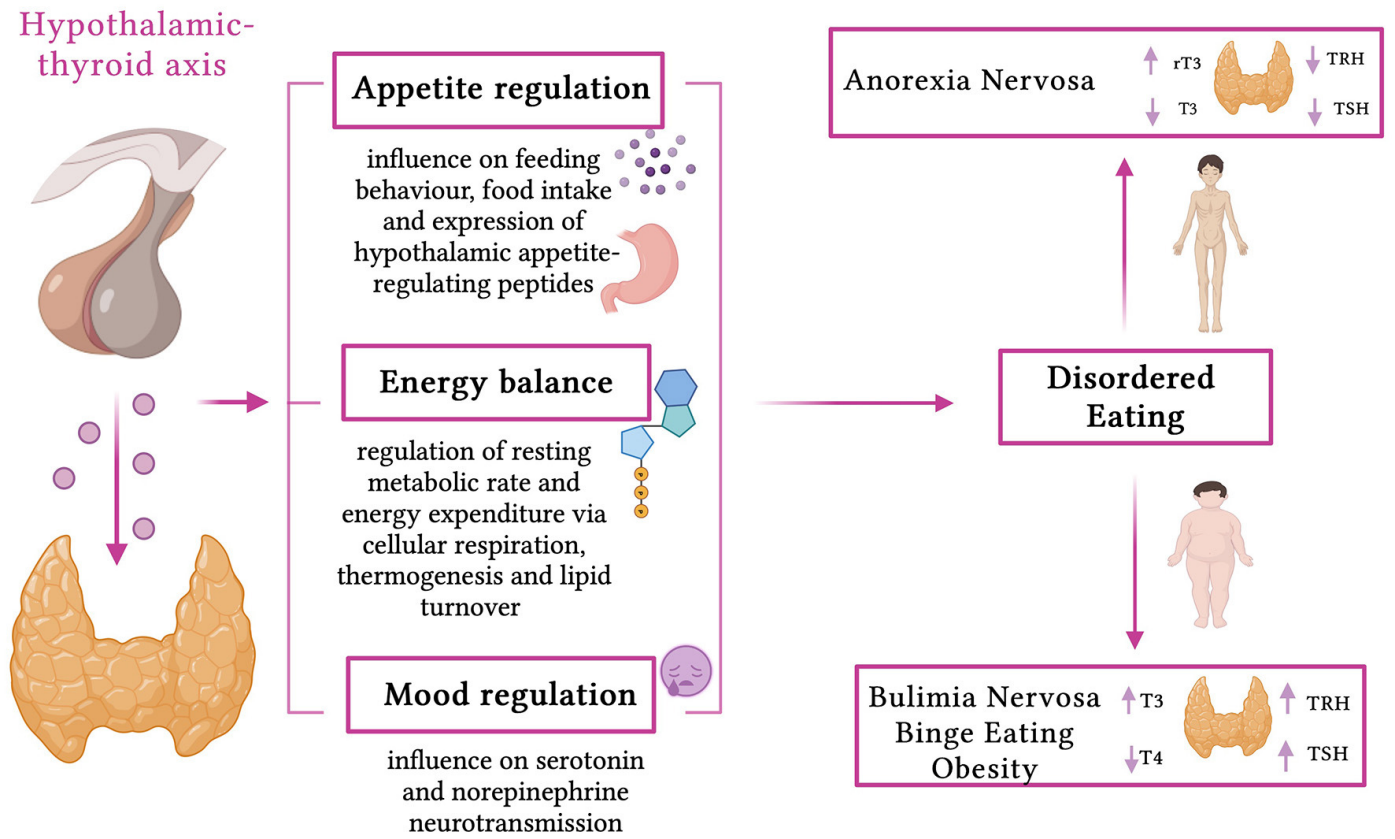
Effects of thyroid hormones and thyroid dysfunction in eating disorders (created with BioRender.com, accessed on April 8, 2023).

Even if obesity in itself is not framed as an eating disorder and has always been considered a separate pathology, obesity and ED are not separate issues. As illustrated in Figure 1, they are intimately connected and could be considered as part of a continuum; it has become evident how one condition can lead to another, such as binge eating disorder (BED) and bulimia nervosa (BN), and that they share the same repercussions in terms of psychosocial, metabolic, and nutritional health (17–19). Additionally, different mechanisms linking obesity with EDs and vice versa have been proposed, among other environmental (family and peer teasing, perceived social pressure, criticism, or bullying) and individual (biological genetic risk factors, low self-esteem, negative self-evaluation, and body dissatisfaction) risk factors (19).

Nutritional and hormonal factors are closely related to the regulation of metabolism in human beings.

The nutritional alterations affect each and every aspect of the functioning of the endocrine glands, leading to serious disorders (20). Plenty of endocrine abnormalities have been described in patients with ED, both undernutrition and overnutrition, but, between them, thyroid dysfunction is often underestimated, especially in pediatric patients. While the relationship between body weight and thyroid status is complex, an increased risk of developing thyroid diseases has been reported in patients with obesity, in patients with ED who are overweight/obese, in those who have disorders such as BN and BED, and in those with underweight conditions such as anorexia nervosa (AN) (21–25). Notably, the thyroid acts as a fundamental regulator of resting metabolic rate and energy expenditure, influencing cellular respiration, thermogenesis, and lipid turnover (26–28). Moreover, thyroid hormones (TH) have effects on appetite regulation, influencing feeding behavior, food intake, and expression of hypothalamic appetite-regulating peptides, and on mood regulation, influencing serotonin and norepinephrine neurotransmission, showing a potentially crucial role in the pathogenesis of the abovementioned disorders (29).

Hence, this narrative review aims to further investigate the hypothalamic-pituitary-thyroid axis in undernourished and overnourished patients with ED, including obesity, in order to highlight the relationship between weight control and thyroid function and its effects, to describe the crucial role of TH monitoring, and to consider therapeutic and preventive strategies in children and adolescents.

## 2. Methods

We present a narrative literature review (30), emphasizing thyroid dysfunction and its consequences in pediatric patients affected by ED, including those who are obese. Reviews, original research papers, metanalyses, clinical trials, and case reports published in the last 15 years up to February 2023 were reviewed. Letters, commentaries, and articles that had no full text accessible in English were not included. We performed a non-systematic search on the PubMed, Scopus, and Web of Science platforms. The following terms (alone and/or in combination) were used for the literature search: eating disorders, anorexia nervosa, binge eating disorder, obesity, nutrition, weight, endocrine, thyroid, euthyroid sick syndrome, hypothyroidism, pituitary-thyroid axis, insulin resistance, therapy, recovery, weight gain, and weight loss. The authors assessed the abstracts (*n* = 130) and reviewed the full texts of relevant articles (*n* = 101) that were analyzed and discussed.

## 3. Thyroid, metabolism, energy balance, and body weight control

Thyroid function plays a crucial role in body weight regulation, mainly through regulating energy expenditure and metabolism (26).

Thyroid hormone receptors are ubiquitous and influence metabolic pathways in virtually all systems throughout an organism's entire life (26). Indeed, historically, the gold standard for evaluating thyroid action was the basal metabolic rate (BMR), measured using indirect calorimetry, until immunoassay evaluation of TH and thyroid stimulating hormone (TSH) replaced this method (26, 27).

Peripheral and central actions are described in the metabolic control of TH (28), including molecular mechanisms of TH action, regulation of the lipid profile, nuclear receptor crosstalk, the impact of corepressors in metabolic regulation, the interaction of TH with adrenergic signaling, influences of TH on thermogenesis, and central regulation of the autonomic nervous system (28).

The effects of severe thyroid dysfunction (both in the case of hyperthyroidism and hypothyroidism) on energy expenditure (EE) are well described (27, 31), but the effects of subtle thyroid dysfunction on EE have not been clearly defined yet, especially in children (26, 29, 31).

Hyperthyroidism (excess TH) results in a hypermetabolic state and increases BMR, leading to weight loss, increased lipolysis, and gluconeogenesis (27, 32). However, hypothyroidism (reduced TH) is associated with hypometabolism, reduced BMR, weight gain, a decrease in lipolysis, and gluconeogenesis (26, 27).

In healthy subjects, variations in serum TSH are associated with body weight change (26, 27, 33). Indeed, subjects with serum TSH levels in the upper quintiles have a higher BMI, and those with serum TSH levels in the lower quintiles have a lower BMI. Thus, T4 treatment (aimed at re-establishing euthyroidism) is associated with weight loss and increased BMR in hypothyroid patients (26, 27).

Interestingly, modestly increased levels of TSH (with a thyroid function within the reference range) have been associated with body weight in both sexes (31). Specifically, Fox et al. (31) performed a cross-sectional and longitudinal study in a community-based sample and evaluated the TSH concentrations (at baseline and follow-up) of 2, 470 participants (31). The authors noted that TSH concentrations were related to body weight and body weight change during the 3.5 years of follow-up (31). Specifically, at baseline, adjusted mean weight increased from 64.5 to 70.2 kg in the lowest to highest TSH concentration quartiles in women and from 82.8 to 85.6 kg in men (31). At the follow-up, mean body weight increased by 1.5 kg in women and 1.0 kg in men (31). Importantly, the increased TSH concentration was positively associated with weight gain in both sexes.

Moreover, recent research has been focusing on the possible association between thyroid hormones and food preferences in children (34); it has long been known that food preferences are established in early childhood and are influenced by several genetic and environmental factors, on which hormones may act as modulators. TH is one of the main regulators of energy expenditure and leptin and adiponectin secretion, thus indirectly influencing food intake. Hormones could also have a crucial role in the hedonic mechanisms of food intake, activating the dopamine reward system (35). Additionally, THs are involved in the regulation of metabolic processes, such as *de novo* gluconeogenesis and liver and adipose tissue lipolysis and lipogenesis, which might be related to taste perception regulation and food preferences (36). Stanikova et al. (34) assessed food preferences and TH levels in 200 children who were overweight or obese and found that higher fT4 levels directly correlate with unbalanced dietary preferences (high protein, high fat, and low fiber foods). The authors suggest that the minor weight loss observed in patients receiving exogenous thyroxine could be due to increased caloric intake despite an increase in resting energy expenditure (34, 37).

These findings underline the importance of thyroid control in weight balance (26, 31). This concept becomes fundamental in the states of undernutrition and overnutrition, as in the case of ED (26, 27, 29, 31, 38).

## 4. Eating disorders in children and adolescents

EDs are psychiatric disorders characterized by maladaptive cognitions and behaviors related to eating and weight control, such as an excessive preoccupation with weight and shape of the body or a frank deviation of the body image leading to voluntary restriction of food intake or the presence of episodes of binge eating (17). EDs represent a serious health issue due to their medical, psychiatric, and nutritional comorbidities and the risk of suicidal attempts. The latest diagnostic criteria for specific eating disorders are reported in the fifth edition of the Diagnostic and Classification Manual of Mental Disorders (DSM-5, APA 2013) and the Eleventh Revision of the International Classification of Diseases (ICD 11) (39, 40). DSM-5 includes definitions of AN, BN, BED, avoidant restrictive food intake disorder (ARFID), other specified feeding or eating disorder (OSFED), and unspecified feeding or eating disorder (UFED) (39). In our review, we mainly focused on AN, BN, and BED, as reported in Table 1.

**Table 1:** Diagnostic criteria of the EDs studied according to the diagnostic and statistical manual of mental disorders, fifth edition (DSM-5)−2013 publication of the American Psychiatric Association (APA) classification and assessment tool (39).

**Eds**	**Diagnostic criteria**
Anorexia nervosa	• Restricting energy intake relative to requirements leads to a significantly low body weight in the context of age, sex, developmental trajectory, and physical health. Significantly low weight is defined as a weight that is less than minimally normal or, for children and adolescents, less than minimally expected. • Intense fear of gaining weight or becoming fat, or persistent behavior that interferes with weight gain, even at a significantly low weight. • Disturbance in the way in which one's body weight or shape is experienced, undue influence of body weight or shape on self-evaluation, or a persistent lack of recognition of the seriousness of the current low body weight. Subtypes: - Restricting type: During the last 3 months, the individual has not engaged in recurrent episodes of binge eating or purging behavior (i.e., self-induced vomiting or the misuse of laxatives, diuretics, or enemas). This subtype describes presentations in which weight loss is accomplished primarily through dieting, fasting, and/or excessive exercise. - Binge-eating/purging type: During the last 3 months, the individual has engaged in recurrent episodes of binge eating or purging behavior (i.e., self-induced vomiting or the misuse of laxatives, diuretics, or enemas).
Bulimia nervosa	• Recurrent episodes of binge eating—an episode of binge eating is characterized by both of the following: - Eating, in a discrete period of time (e.g., within any 2-h period), an amount of food that is definitely larger than what most individuals would eat in a similar period of time under similar circumstances. - A sense of lack of control over eating during the episode (e.g., a feeling that one cannot stop eating or control what or how much one is eating). • Recurrent inappropriate compensatory behaviors to prevent weight gain, such as self-induced vomiting; misuse of laxatives, diuretics, or other medications; fasting; or excessive exercise. • On average, binge eating and inappropriate compensatory behaviors occur at least once a week for 3 months. • Self-evaluation is unduly influenced by body shape and weight. The disturbance does not occur exclusively during episodes of anorexia nervosa.
Binge eating disorder	• Recurrent episodes of binge eating. An episode of binge eating is characterized by the following: - Eating in a discrete period of time (e.g., within any 2 h) means eating an amount of food that is larger than what most people would eat in a similar period of time under similar circumstances. - A sense of lack of control over eating during the episode (e.g., a feeling that one cannot stop eating or control what one is eating). • The binge-eating episodes are associated with three (or more) of the following: - Eating much more rapidly than normal. - Eating until feeling uncomfortably full. - Eating large amounts of food when not feeling physically hungry. - Eating alone because one feels embarrassed by how much one is eating. • Feeling disgusted with oneself, depressed, or very guilty afterward. • Marked distress regarding binge eating is present. • The binge eating occurs, on average, at least once a week for 3 months. • Binge eating is not associated with the recurrent use of inappropriate compensatory behavior as in bulimia nervosa and does not occur exclusively during the course of BN or AN

Anorexia nervosa (AN) is the most recognized eating disorder, characterized by starvation, intentional and severe weight loss, and malnutrition. A high incidence of coexisting psychiatric conditions, reluctance to seek treatment due to a psychological attachment to maintaining the disorder, and high rates of medical and endocrinological comorbidities are other common features (5, 6, 41, 42). Without early effective treatment, the course is protracted with physical, psychological, and social morbidity and high mortality, accounting for the majority of deaths caused by eating disorders (41, 43, 44). First recognized in France in 1874, the definition of AN has changed significantly over time. According to the DSM-5, for a person to be diagnosed with AN, they must display three key features, which are summarized in Table 1 (39, 45); notably, the previous D criterion of the DSM-IV, related to amenorrhoea, was removed, allowing for the inclusion of a larger population of patients, including premenarchal girls, boys and men, and girls on contraceptives (39, 46). Moreover, no standardized weight loss assessment is currently required for the diagnosis; in DSM-5, they refer to a weight that is less than minimally expected with reference to the Body Mass Index for age percentiles. In adults, a BMI of 18.5 kg/m^2^ is proposed as the lower limit of normal body weight, whereas in children and adolescents, a BMI lower than the 10th percentile is generally used in defining AN (39). The DSM-5 also describes two designated subtypes of anorexia nervosa: the restricting subtype, in which patients achieve weight loss primarily through dietary restriction, fasting, and excessive exercise; and the binge-eating and purging subtype, in which restriction is accompanied by binge eating, purging, or both (39). The two conditions may progress from one subtype to another, generally from the restricting subtype to the binging–purging subtype (8).

Similar to AN, fear of fatness and attempts to lose weight are core symptoms of bulimia nervosa (BN), which is characterized by recurrent episodes of binge eating—uncontrolled eating of an abnormally large amount of food—followed by compensatory behaviors such as self-induced vomiting, purges, or excessive exercise (45). Remarkably, in DSM-5, the BN subtypes purging and non-purging were removed, whereas a new classification regarding the severity of presentation based on the frequency of the episodes was introduced (39).

Nevertheless, the major change from DSM-IV to DSM-5 is the formal integration into a clinical diagnosis of BED, defined as the recurrence of episodes of consumption of a large amount of food with a sense of loss of control over eating and the absence of compensatory behaviors (39, 46, 47). In addition to the binge episodes, individuals meeting BED criteria must exhibit other features related to eating behaviors, as reported in Table 1 (39, 47). In contrast to binge eating in bulimia nervosa, BED occurs without compensatory behavior to eliminate calories consumed. Hence, it is commonly associated with being overweight or obese (6, 47, 48). In both conditions, the episodes must occur at least once a week for an average of 3 months.

Diagnosing BN and BED in childhood and adolescence could be challenging due to the difficulty of objectively assessing what is considered a ‘large intake of food' during different life stages and because diagnosis mostly relies on self-reporting and describing the episodes. Moreover, the difficulty in expressing the sense of loss of control could make diagnosing and estimating the prevalence of BED in childhood challenging. Nevertheless, BN and BED appear to be the most prevalent eating disorders, with a lifetime prevalence in adolescents and young adults ranging from 0.8 to 2.6% in BN and 0.6 to 6.1% in BED, and from 0.1–0.16% in BN and from 0.3 to 0.7% in BED, respectively, in girls and boys (1, 48–50). In addition, BEDs of lower frequency and/or limited duration are more prevalent.

The onset of ED usually occurs during adolescence and young adulthood, with the peak of incidence between 13 and 19 years for AN and BN and a bimodal distribution in early adolescence and early adulthood in BED (51). Nevertheless, the age of onset of juvenile ED has decreased during the last decades, and ED may also have onset in children as young as 5 to 12 years old, a rising concern given the noted elevation in psychiatric and medical morbidity in younger patients with ED (2, 52–55).

Epidemiological estimates in children and adolescents are quite complex due to the lack of uniformity in studies and the modification of diagnostic criteria over time. Furthermore, epidemiological data change according to geographic location and sex. Traditionally, prevalence studies of eating disorders have focused on high-income Western countries, showing an overall DSM-5 ED lifetime prevalence among female adolescents and young women between 5.5 and 17.9%, respectively, and among male adolescents and young men between 0.6 and 2.4%, respectively (1). Eating disorders in men appear to be 10 times less common, but they are believed to be grossly underestimated due to how EDs are assessed and treated, largely reflecting a female-oriented diagnostic framework as EDs are among the most gendered of psychiatric illnesses (56). Moreover, while challenging the assumption that ED is a phenomenon that mainly affects Western female populations, recent studies have suggested that eating disorders also appear to be a significant global concern (57, 58). Disordered eating attitudes have promptly emerged in areas where societies are in transition and where large social and demographic changes are occurring, such as in the Middle East and North Africa, as well as in Eastern Europe, Asia, and Latin America (1, 7, 50, 59, 60). This increase can be attributed to many factors, including a better understanding of the pathological conditions, paradigm changes in the expectations people have about their bodies (e.g., body image dissatisfaction when compared to Western fashion), increased demand for mental healthcare, improvements in healthcare systems, and the adoption of a more modern lifestyle, leading to changes in the perception of the “ideal body” (50). In addition, after the COVID-19 pandemic, ED epidemiology changed. Taking into account the well-known effects on mental health status as well as on eating behaviors that the pandemic and the lockdown had on the general population, with even more impact on young people, it is not surprising that the majority of most qualitative studies report a worsening of ED symptoms in both AN, BED, BN, and OFSTED patients (61). A recent review showed a worryingly average increase of 83% in pediatric admissions (61). Furthermore, the increase in prevalence in the last decades could be related to the increase in vulnerable populations, such as children with obesity, as both BN and BED are more likely to occur in overweight populations, as well as gender-diverse youth, where disordered eating develops as a maladaptive coping strategy to manage minority stress and to conform to community-specific standards of attractiveness (62–65).

## 5. Hypothalamic-pituitary-thyroid axis in underweight/starved ED patients (AN)

AN is a devastating disease, and its consequent undernutrition state was shown to have negative effects on multiple endocrine axes, such as gonadal, adrenal, and thyroid ones (66–68). Moreover, this eating disorder affects growth hormone, insulin-like growth factor-1, and various adipokines, such as leptin, ghrelin, peptide YY, and amylin (66). One of the endocrine systems negatively affected by AN is the hypothalamic-pituitary-thyroid axis. Although it has not been extensively studied, especially in pediatric and adolescent patients, this axis is profoundly affected by AN and starvation (66). Patients with AN often show typical hypothyroidism symptoms, such as hypothermia, hypotension, dry skin, reduced metabolic rate, delayed Achilles reflex half-relaxation time, and, importantly, bradycardia (66). Moreover, from a biochemical point of view, patients with AN have thyroid hormone abnormalities compatible with the so-called “sick euthyroid syndrome” or “non-thyroidal illness syndrome (NTIS)” (69, 70).

NTIS is characterized by low triiodothyronine (T3), low to normal thyroid stimulating hormone (TSH), and increased reverse triiodothyronine (rT3) levels. These changes are assumed to be a response to systemic illness, both acute and chronic, such as severe malnutrition, through different mechanisms (70–72). Interestingly, Selvaraj et al. (73) hypothesized that sick euthyroid syndrome is a protective defensive mechanism that lowers the metabolic rate in response to the oxidative stress of acute illness (73).

In any event, the alterations observed in this syndrome involve thyroid activity at various levels. Specifically, the main changes are observed in the activity of the iodothyronine deiodinase, in the secretion of TSH and thyrotropin-releasing hormone (TRH), in the ability of thyroid hormone to bind plasma proteins, and in its transport to peripheral tissues (70, 71, 73).

These thyroidal abnormalities are considered a continuum of changes depending on the severity and time course of the illness and are part of a coordinated reaction involving the immune and endocrine systems (74). Importantly, as underlined by Lee and Farwell (70) and confirmed by Warner and Beckett (74), the alterations observed in this condition are not limited to thyroid hormones but are often accompanied by changes in other endocrine systems, such as increases in serum ACTH and cortisol levels and decreases in serum gonadotropin and sex hormone concentrations, as observed in malnourished patients (70, 74). Moreover, when discussing EDs, it is worth underlining that the abnormalities of NTIS are not limited to organic illness but also manifest in acute psychiatric pathologies (75–77).

A deep description of the potential underlying mechanisms for the thyroid hormone abnormalities in NTIS is beyond the scope of our review, but the main mechanisms involved in this condition are concisely reported hereafter:

- **Alterations in iodothyronine deiodinases**. Iodothyronine deiodinases are enzymes that catalyze the sequential monodeiodination of the iodothyronines; their role is to activate or deactivate thyroxine (T4) (78). There are three iodothyronine deiodinases. Type 1 (D1) and type 2 (D2) catalyze the activating reaction, converting T4 to T3 through the removal of outer ring iodine). In contrast, type 3 (D3) catalyzes the deactivating reaction, converting both T4 and T3 to T3 by removing inner ring iodine) (70, 74, 78).
- **Alterations in thyroid-stimulating hormone secretion**. The decrease in TSH secretion in NTIS seems to be correlated both to an increased D2 in the pituitary and at hypothalamic levels (leading to a local production of T3 and a decreased TSH synthesis) and to a decreased TRH production (74). Importantly, the TRH and TSH decrease seem to be mediated by leptin, a hormone encoded by the *OB* gene and secreted by adipocytes (74). Indeed, leptin has been reported to directly regulate TRH production, and leptin levels are directly correlated with TSH levels (70, 79). Importantly, serum leptin decreases during fasting and malnutrition, as in patients with AN (67, 68, 74, 79, 80). These changes seem to be a sort of adaptive mechanism that functions to reduce energy expenditure and catabolic processes (66, 70).
- **Alteration in serum thyroid hormone-binding proteins**. The majority of thyroid hormone in plasma is bound to various binding proteins, such as thyroxine-binding globulin (TBG), transthyretin, and albumin (69, 70). Typically, the serum levels of binding protein are decreased in a state of malnutrition or high catabolism, as in AN (66, 68).
- **Alterations in the thyroid hormone transporter**. In NTIS, impaired transport of T4 into peripheral tissues such as the liver and kidney was shown, as in starvation (66, 68). This leads to a decrease in the availability of the substrate for T3 production (70, 81). The underlying mechanisms of altered thyroid hormone transport in this context have not been clarified yet. Thus, further studies are needed.

### 5.1. Treatment of thyroid dysfunction in patients with AN

As the illness resolves or the patients recover from the malnutrition state, the alterations in thyroid hormone concentrations tend to normalize (74, 81–83). During the recovery phase, a modest increase in serum TSH levels can be seen (69, 70). Indeed, full recovery with the restoration of normal levels of thyroid hormone may take weeks or months (69, 71).

The clinical significance of the thyroid abnormalities observed in the euthyroid sick syndrome has not been understood yet, and this has resulted in conflicting data concerning the effects of treatment with thyroid hormone on clinical outcomes (69, 70, 73). Indeed, if the changes typical of NTIS are a physiologic adaptive mechanism for decreased metabolism, replacement therapy may not be necessary or even harmful (70, 84). However, if these changes are considered pathologic, treatment may be beneficial and thus indicated (70).

Onuer et al. evaluated 28 women with AN and 49 healthy controls and identified a correlation between low T3 levels and low resting energy expenditure (84). Moreover, the authors followed a subset of 17 subjects with AN during their weight gain. They observed a significant correlation between rising T3 levels and increased resting energy expenditure (*r* = 0.78, *P* < 0.001), suggesting a key role for T3 in modulating metabolic rate (68, 84).

As current evidence suggests that hormonal imbalances observed in NTIS are a combination of physiologic adaptation and pathologic alteration, there is no persuasive evidence for the use of thyroid hormone replacement in patients with NTIS (74). Indeed, treatment and management of underlying medical illnesses are the main focus. Moreover, thyroid hormone supplementation has not been studied in patients with AN, and different reviews recommend against this strategy, both for the potential adverse effects on weight (weight loss and muscle wasting) or arrhythmias (68, 70) and for the realistic risk of abuse, as shown by Woodside et al. in an interesting case report and more recently reported by Neudahina et al. (75, 85, 86). The desire to lose weight by subjects with AN is often associated with maladaptive behaviors, including drug abuse such as diuretics, laxatives, hypoglycaemic drugs, and hormonal drugs, such as thyroid hormone replacement therapy, that enhance the metabolism, leading to weight loss (86–88).

Interestingly, Stoving et al. (89) evaluated via ultrasonography the thyroid gland in 22 patients with AN and 44 age- and sex-matched control subjects (89). The authors highlighted that thyroid volume in patients with AN was markedly reduced compared to the controls and also with respect to the volume expected from age and body weight. This result indicates thyroid atrophy, considered part of the vicious cycle of maintaining anorectic or depressive symptomatology. Indeed, the hypothalamic-pituitary-thyroid axis has a known role in mood regulation; thus, alterations in this axis, as observed in patients with AN and NTIS, may be at least partially responsible for the coexisting depressive symptoms in these subjects (75, 76, 90). Wronski et al. (75), aiming to investigate the associations between pituitary-thyroid function and psychopathology (in particular depressive symptoms) at different stages of AN, performed a combined cross-sectional and longitudinal study design (75). The authors assessed pituitary-thyroid status (free T3, free T4, conversion ratio FT3/FT4, and TSH) in 77 young acutely underweight women with AN (acAN) and in 55 long-term weight-recovered individuals with former AN (recAN) in a cross-sectional comparison to 122 healthy controls. In addition, the pituitary-thyroid status of 48 acAN patients was reassessed after short-term weight restoration. The researchers also executed correlation analyses of pituitary-thyroid parameters with self-reported measures of psychopathology. The study underlined that lower FT3 concentrations and FT3/FT4 ratios were associated with more severe depressive symptoms in acAN. Moreover, associations between conversion ratios FT3/FT4 and psychopathology persisted in short-term weight restoration. Although the study's findings might open doors to the use of low-dose thyroid hormone supplementation in certain patients with AN (for instance, the one showing more severe psychiatric impairment), further research is needed to have more consistent results.

## 6. Hypothalamic-pituitary-thyroid axis in ED patients with overweight/obesity (BN, BED)

Traditionally, eating disorders and obesity have been considered separate pathologies. In recent years, nevertheless, it has become clear how these conditions share the same repercussions in terms of psychosocial, metabolic, and cardiovascular health. It has also been demonstrated how obesity may lead to EDs and vice versa, especially when considering the unrealistic beauty standards imposed by Western society. The promotion of self-objectification, especially through channels such as social media, often overexposes children and adolescents to these standards (19). It has been widely recognized that BN and BED may lead to becoming overweight and obese, triggering a vicious circle of unsatisfying body image and frustration that may induce compulsory food intake (91).

Hypothyroidism is one of the most common endocrine disorders in the general population, with an estimated prevalence of subclinical disease up to 10%, which is thought to be even higher in the population with obesity (92, 93).

Hypothyroidism has been known for centuries to contribute to being overweight due to fatty tissue accumulation, decreased resting energy expenditure, thermogenesis, reduced physical activity, and subcutaneous edema due to glycosaminoglycan deposition (94).

In recent decades, research has focused on thyroid axis impairment as a consequence of obesity rather than its cause. Indeed, overeating has been associated with hypothalamic-pituitary-thyroid axis activation and changes in thyroid function, as summarized in Table 2 (22, 97).

**Table 2:** Thyroid function alterations in patients who are overweight.

**Altered pathway**	**Mechanism**	**Results**
Leptin – inflammatory cytokines (95)	Direct hypothalamic stimulation	TRH secretion
Fat tissue deiodinase (92)	T4 conversion to T3	enhanced T3 action on peripheral tissues
Adipose cell stimulation by thyroid hormones (28)	Leptin secretion	Enhanced hypothalamic stimulation
Glucose metabolism (96)	Thyroid hormones stimulate gluconeogenesis and intestinal glucose absorption Thyroid hormones directly activate pancreatic B cells	Insulin hypersecretion and insulin resistance

Several studies have reported slightly elevated TSH and T3 levels and slightly lower T4 levels in up to 23% of overweight subjects, mostly below the cutoff for subclinical hypothyroidism (98, 99). Leptin and inflammatory cytokines have been hypothesized to possibly be responsible for direct hypothalamic activation leading to TRH secretion, and fat tissue deiodinase is thought to play a role in increasing the levels of T3, possibly as an adaptive mechanism to increase basal metabolism in overweight subjects or as a response to inflammatory cytokines secretion by the adipose tissue (92, 95). A stimulating effect of thyroid hormones on adipocytes has also been reported, leading to increased leptin secretion (28, 100). The observation that imbalances in thyroid function in obesity were more often due to adaptive mechanisms than primary thyroid disease led to the hypothesizing of a new clinical entity different from subclinical hypothyroidism called “hyperthyrotropinemia” (101, 102).

European Endocrinology Guidelines published in 2020 recommend thyroid function screening in patients with obesity by measuring serum TSH; the authors highlight the importance of distinguishing between TSH augmentation due to autoimmune hypothyroidism and fat-induced hyperthyrotropinemia and do not suggest routine assessment of fT3 because its levels in subjects with obesity often depend on peripheral conversion due to non-thyroidal illness (103).

Additionally, thyroid hormones affect glucose metabolism to control weight, particularly in subjects with excess weight. The action differs between skeletal muscle, where insulin promotes glucose intake, and the liver, where this action is inhibited, influences insulin resistance (104).

In the prenatal and neonatal periods, TH enhances beta-pancreatic cell development and function; on liver tissue, TH increases GLUT2 expression on the cellular surface, with a consequential increase in glucose output through gluconeogenesis and glycogenolysis; on the gastrointestinal tract, TH increases glucose absorption. Chronically, this leads to insulin hypersecretion and glucose intolerance; TH directly stimulates pancreatic insulin secretion (105).

A recent population study on adult subjects evaluated glucose intolerance through an oral glucose tolerance test and thyroid function evaluation to estimate thyroid function in different statuses of glucose intolerance; the results showed a higher incidence of glucose metabolism impairment in patients with subclinical hypothyroidism (106).

A recent study compared insulin-fasting levels and the homeostatic model assessment (HOMA) index between hypothyroid children with obesity and those with normal weight, with the addition of a healthy control group; the results showed higher leptin and insulin-fasting levels and a higher HOMA index in both hypothyroid children with obesity and normal weight, with higher values in the population with obesity compared to healthy controls. Adiponectin levels were found to be lower in children with obesity compared to the lean hypothyroid children and the healthy ones. IR in hypothyroidism is believed to originate from an altered expression of glucose transporters on cellular surfaces; this study suggests an additive impact of obesity and hypothyroidism on glucose metabolism (106).

Adult studies found an increased risk of type 2 diabetes, metabolic syndrome, and cardiovascular events in patients with subclinical hypothyroidism (TSH levels in the upper-normal range with normal thyroid hormones) and, conversely, a higher level of T3 and T4 in insulin-resistant patients and patients with diabetes compared to healthy controls (107, 108). Subclinical hypothyroidism has been associated with a predisposition to non-alcoholic fatty liver disease in children and adolescents with obesity (109, 110).

Based on these findings, it has been hypothesized that the association between obesity, insulin resistance, and hyperthyrotropinemia may be due to a generalized (both central and peripheral) resistance to thyroid hormones driven by increased caloric intake and adipose tissue deposition (104).

Further studies are needed to evaluate the effect of exogenous thyroxine on insulin resistance in children with obesity and hyperthyrotropinemia.

To conclude, it is worth mentioning a recent study by Staníková et al. (34) that showed a possible link between free thyroxin levels and dietary preference for foods rich in fat and protein. Indeed, the researchers examined the interrelations between food preference and peripheral thyroid activity in 99 non-obese and 101 obese children and adolescents selected from the patients of the Obesity and Metabolic Disease Out-patient Research Unit at the National Institute for Children's Diseases in Bratislava. The researchers found that higher serum levels of FT4 were linked with higher AST and ALT levels in obese children and adolescents and that FT4 was also the best predictor of preference for foods rich in fat and low in fiber. This may indicate that FT4 could contribute to the development of childhood obesity by modulating food preferences, and, on the contrary, lower FT4 could have a protective effect, acting against the development of overweight conditions (34).

### 6.1. Treatment of thyroid dysfunction in patients with obesity and the effect of weight loss

Subclinical hypothyroidism has a prevalence of 7–23% in the overweight/obese population. International guidelines and recent literature recommend using the same reference ranges and target TSH levels for the treatment of hypothyroidism in patients with obesity as for normal-weight subjects (103, 111, 112).

Treatment of overt hypothyroidism, defined as TSH > 10 mU/L, is recommended; there is an ongoing debate regarding the treatment of subclinical hypothyroidism, defined as TSH between 4.5 and 10 mU/L with normal T4. No specific recommendation can be made due to the lack of data on the pediatric population and the uncertainty associated with treating subclinical hypothyroidism in adult patients (93).

European guidelines recommend that the decision to treat hyperthyrotropinemia with thyroid hormones takes into account several factors, such as the coexistence of autoantibodies or other causes of primary hypothyroidism (103).

Administration of levothyroxine in patients with obesity and hypothyroidism has been extensively studied, but several authors report only a minor effect on weight loss in these subjects (113).

Treatment of obesity with the administration of thyroid hormones in the absence of thyroidal illness is currently not recommended due to the mild effect on weight loss compared to the increased cardiovascular risk (103, 114). Administration of levothyroxine is only recommended in patients with obesity and a diagnosis of hypothyroidism (93).

Weight loss leads to a rapid reduction of serum TSH and fT3, suggesting that dysthyroidism in obesity is a reversible condition; this supports the recommendation of not routinely treating hyperthyrotropinemia in obesity and the hypothesis that thyroidal dysfunction is considered a consequence of hypothyroidism (29, 93, 99). Ecographic alteration of thyroid tissue in the absence of autoantibodies (seronegative thyroiditis) reported in a subgroup of patients with obesity often normalizes following weight loss in the absence of specific hypothyroidism therapy (93, 115).

## 7. Conclusions

A strong relationship between thyroid function and body weight control is described. Thyroid alterations occur in underweight and overweight patients with ED and represent a continuum of changes depending on the severity and time of the disease course involving the endocrine system and metabolic processes. In several conditions, the disorders are mainly a consequence rather than the cause of thyroid dysfunction, and they are reversible during the control of the ED course.

Considering the relevant role TH plays not only in EE but also in metabolic control and cardiovascular risk related to dysmetabolism and mood regulation, continuous monitoring of thyroid homeostasis in a patient with ED is mandatory to prevent severe organ dysfunction and psychological complications and to start early treatment when necessary.

## Author contributions

VCa, VM, FS, CH, and GZ: conceptualization. VCa, VM, FS, CH, MB, VCo, GM, SR, PV, and GZ: methodology. VCa, VM, FS, CH, MB, VCo, and GM: writing—original draft preparation. VCa, VM, SR, PV, and GZ: writing—review and editing. VCa, SR, PV, and GZ: supervision. All authors contributed to the article and approved the submitted version.
